# Variations in the Sporulation Efficiency of Pathogenic Freshwater Oomycetes in Relation to the Physico-Chemical Properties of Natural Waters

**DOI:** 10.3390/microorganisms10030520

**Published:** 2022-02-27

**Authors:** Dora Pavić, Dorotea Grbin, Marija Gregov, Josip Ćurko, Tomislav Vladušić, Lidija Šver, Anđela Miljanović, Ana Bielen

**Affiliations:** Faculty of Food Technology and Biotechnology, University of Zagreb, Pierottijeva 6, 10000 Zagreb, Croatia; dpavic@pbf.hr (D.P.); dorotea.polo@gmail.com (D.G.); marija.gregov@pbf.unizg.hr (M.G.); jcurko@pbf.hr (J.Ć.); tvladusic@pbf.hr (T.V.); lsver@pbf.hr (L.Š.); amiljanovic@pbf.hr (A.M.)

**Keywords:** *Aphanomyces astaci*, crayfish plague, dissolved organic carbon (DOC), humic acid (HA), *Saprolegnia parasitica*, saprolegniosis, spectral absorption coefficient (SAC), zoospores

## Abstract

Oomycete pathogens in freshwaters, such as *Saprolegnia parasitica* and *Aphanomyces astaci*, are responsible for fish/crayfish population declines in the wild and disease outbreaks in aquaculture. Although the formation of infectious zoospores in the laboratory can be triggered by washing their mycelium with natural water samples, the physico-chemical properties of the water that might promote sporulation are still unexplored. We washed the mycelia of *A. astaci* and *S. parasitica* with a range of natural water samples and observed differences in sporulation efficiency. The results of Partial Least Squares Regression (PLS-R) multivariate analysis showed that SAC (spectral absorption coefficient measured at 254 nm), DOC (dissolved organic carbon), ammonium-N and fluoride had the strongest positive effect on sporulation of *S. parasitica*, while sporulation of *A. astaci* was not significantly correlated with any of the analyzed parameters. In agreement with this, the addition of environmentally relevant concentrations of humic acid, an important contributor to SAC and DOC, to the water induced sporulation of *S. parasitica* but not of *A. astaci*. Overall, our results point to the differences in ecological requirements of these pathogens, but also present a starting point for optimizing laboratory protocols for the induction of sporulation.

## 1. Introduction

Animal pathogenic oomycetes are a cause of serious diseases worldwide [1,2,3], and in freshwater ecosystems species from the genera *Aphanomyces* and *Saprolegnia* are the most significant, since they cause severe disease outbreaks both in aquaculture and in the wild [4,5,6,7]. Crayfish plague, a disease caused by *Aphanomyces astaci* is responsible for decimating the populations of indigenous crayfish species in Europe [8] and elsewhere (e.g., Asia and South America) [9,10,11,12]. The pathogen was introduced into Europe along with North American non-indigenous invasive crayfish species which now act as its carriers, being mostly resistant to infection due to a long co-evolution with the pathogen [13,14]. Members of the genus *Saprolegnia* cause saprolegniosis, which is responsible for significant economic losses in salmonid farms and hatcheries [15,16,17,18]. Among them, *Saprolegnia parasitica* is highly virulent and widespread [4,7,19,20], and recent studies suggest that aquaculture facilities can act as the sources of its spread into the natural environment [21,22].

The complete life cycle of oomycetes involves asexual and sexual stages, although in some species, including *A. astaci*, the sexual stage has not been documented [6,23,24]. The asexual stage is considered crucial for pathogen’s dispersal, since it includes the production of motile zoospores, the major infectious stage of the life cycle [23,24]. Zoospores are released into the surrounding water from the hyphal tips (i.e., sporangia) after colonizing the infected host tissue [23,25], and the new infection is established when they locate the suitable host by chemotaxis [26,27]. Zoospores encyst on the surface of the host, such as on the crayfish cuticle or fish skin, and then germinate into hyphae that penetrate the body of the host [6,28,29,30]. In some oomycete species, including *A. astaci* and *S. parasitica*, if zoospores are unable to find a suitable host, they encyst and then release a new zoospore generation (repeated zoospore emergence), thereby increasing the possibility of finding a suitable host [31,32]. Dispersal of zoospores from one host to another by water and by contaminated items (like fishing gear) present the main way of spreading the oomycete diseases in freshwater systems [25,33,34].

The formation of zoospores can be triggered under laboratory conditions, for example, by the lack of nutrients or after a sudden drop in temperature [28,35,36]. Most protocols for sporulation of oomycetes are therefore based on washing the mycelium with stream or lake water, but without defining the components present in the water [32,37,38]. During our work with *A. astaci* and *S. parasitica*, we noticed that sporulation efficiency varied depending on the water used to wash the mycelium. This suggested that the water composition might influence the sporulation process, but there is little data on this in the literature. Several in vitro studies have shown that some salts, such as KCl, NaCl, MgCl_2_, CaCl_2_ or K_2_SO_4_, can affect the formation, motility and germination of oomycete zoospores [31,39,40], but the salt concentrations used were significantly higher than in natural freshwaters [41]. However, a recent study used environmentally relevant concentrations of a sea salt mixture ranging from 0 to 45 g/L and found that survival, growth and infectivity of the plant oomycete pathogen *Phytophthora ramorum* were negatively correlated with salt concentration [42]. At concentrations > 20 g/L, no zoospores were released, but infection could still occur via mycelial growth.

We hypothesized that physico-chemical characteristics of natural waters can affect the intensity of sporulation of freshwater pathogenic oomycetes, and the aim of this study was to test for the first time the sporulation efficiency of two oomycete pathogens, *A. astaci* and *S. parasitica*, upon washing their mycelia with natural surface waters of varying physico-chemical characteristics. The subsequent statistical analysis allowed us to identify the specific parameters of natural waters that could stimulate or inhibit the zoospore formation in freshwater oomycetes.

## 2. Materials and Methods

### 2.1. Water Sampling

**Figure 1:**
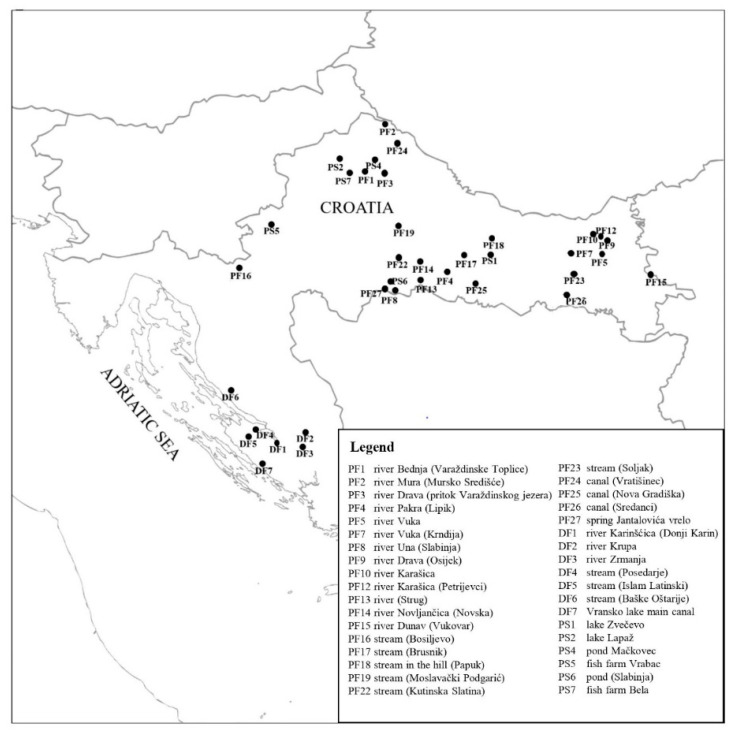
Locations of water sampling (*n* = 36) in Croatia. P—Pannonian ecoregion, D—Dinaric ecoregion, F—flowing, S—stagnant.

Water sampling was performed during winter 2018/2019 in different freshwater bodies in Croatia (Figure 1, Table S1). Water sampling locations (*n* = 36) were selected to vary in terms of geological composition of the substrate, ecosystem type (lentic or lotic) and ecoregion (Dinaric with continental or Mediterranean climate, or Pannonic with continental climate) (Table S1). For some of the locations, data on the presence/absence of the model pathogens *A. astaci* and *S. parasitica* were also available. Water was sampled into autoclaved 1 L polyethylene bottles previously washed three times with the sample and kept in the dark and on ice during transport. Upon arrival to the laboratory, the water samples were immediately autoclaved and then placed in a dark room at +18 °C until physico-chemical analyses (see Section 2.2) and sporulation experiments (see Section 2.3). Noteworthy, water had to be autoclaved before the sporulation experiments to exclude the activity of aquatic microbial communities as a factor that could influence sporulation intensity. Since autoclaving was expected to change the water composition compared to the original sample at the sampling site (e.g., phosphates may form insoluble salts with bivalent metals and precipitate, CO_2_ may be lost due to heating, etc.), all physico-chemical analyses of the water were performed after autoclaving.

### 2.2. Physico-Chemical Analyses of Water

Physico-chemical parameters of the autoclaved water samples, i.e., spectral absorption coefficient (SAC), dissolved organic carbon (DOC), pH, electrical conductivity (EC), ammonium nitrogen (NH_4_-N), nitrate nitrogen (NO_3_-N), SO_4_^2−^, F^−^, Cl^−^, Mg^2+^, Ca^2+^, Br^−^, total nitrogen (TN) and total phosphorus (TP), are listed in Table S2. Electrical conductivity and pH were measured using a pH/Cond—meter inoLab 720 instrument (WTW, Xylem Analytics, Weilheim, Germany). The concentration of TP in water samples was determined according to ISO 6878_2004 (DIN EN 6878/D11), while TN was determined according to EN ISO 11905-1. Ammonium nitrogen (NH_4_-N) was determined according to ISO 7150-1 (DIN 38406 E5-1, UNI 11669:2017). SAC was measured at 254 nm using a DR 6000 Spectrophotometer (Hach, Düsseldorf, Germany) according to DIN 38404 Part 3 (C3). DOC was determined using TOC—LCPH FA E200 (Shimadzu, Kyoto, Japan). Dissolved anions (fluorides, chlorides, nitrates, sulfates) and cations (calcium, magnesium) were determined according to HRN EN ISO 10304-1: 2009/cor.1:2012 and HRN EN ISO 14911:2001 on the ion chromatograph DIONEX DX-500 with a conductometric detector (CD20) in combination with electrochemical suppressor. An anion column Dionex IonPac AS9-HC and a cation column Dionex IonPac CS12A were used. In addition, 9 mmol/L Na_2_CO_3_ was used to elute the anions and 20 mmol/L methanesulfonic acid was used to elute the cations. The eluent flow rate through the column was 1 mL/min.

### 2.3. Sporulation

For the in vitro sporulation experiments, we used *Aphanomyces astaci* isolate PEC 8 (haplogroup B), provided by F. Grandjean (University of Poitiers, Poitiers, France), and *Saprolegnia parasitica* isolate A1, collected from the surface of eggs of rainbow trout *Oncorhynchus mykiss* (Walbaum, 1792) [21]. Both isolates were cultured and maintained in the laboratory at 18 °C on the appropriate solid growth media: peptone-glucose (PG1) medium [43] for *A. astaci*, and glucose-yeast (GY) medium [44] for *S. parasitica*.

To test for the differences in sporulation efficiency after washing the mycelia with different samples of natural waters, previously described sporulation protocols were used [32,37], with some modifications. Namely, uniform pieces of agar (4 mm^2^) containing the fresh mycelial tips of pure cultures were cut and placed into wells of 12-well plates filled with 3 mL of liquid PG1 medium for *A. astaci* or liquid GY medium for *S. parasitica*. Cultures were incubated at 18 °C for four days for *A. astaci* and two days for *S. parasitica*. In the case of *A. astaci*, after three days of incubation the agar plug was removed, and grown hyphae were placed back in the same well filled with liquid PG1 medium and then left to grow for one more day. After incubation of both pathogens, the liquid medium was removed, and hyphal biomass was washed three times with 2 mL of autoclaved natural water samples using sterile Pasteur pipette and then left in 4 mL of the same autoclaved water for 24 h at 18 °C. Then, the mycelia were removed, and the produced motile zoospores were counted in the Thoma chamber using a light microscope LCD MICRO 5MP (Bresser, Rhede, Germany). Three biological replicates (i.e., mycelial inoculums originating from three independently grown cultures) were made for each water sample (i.e., location) and oomycete species, and the number of zoospores of each replicate was counted three times. The average number of zoospores/mL for each location is given in Table S2.

To test whether the addition of humic acid (HA), as an ubiquitous substance that contributes to both SAC and DOC, can induce sporulation of *A. astaci* and *S. parasitica*, a range of environmentally relevant HA concentrations were added to the water samples. Two types of water were selected for this experiment: artificial water (AW, ISO 7346-1 and 7346-2, ISO 1996) and one of the natural water samples from our dataset (PF4, Table S2). Artificial water (AW) was prepared by adding salts to Milli-Q water according to ISO 7346-1 and 7346-2 (ISO 1996), i.e., 294.0 mg/L CaCl_2_ × 2 H_2_O, 123.3 mg/L MgSO_4_ × 2 H_2_O, 63.0 mg/L NaHCO_3_ and 5.5 mg/L KCl, and autoclaved. HA stock solution (160 mg/L in 0.1M NaOH, DOC = 48.0 mg/L, SAC = 379.9 m^−1^) was filtered through a syringe polyester filter (pore size = 0.45 µm, d = 25 mm, CHROMAFIL^®^ Xtra PET-45/25, Macherey-Nagel, Düren, Germany). Water samples with different concentrations of HA, i.e., 4, 8 and 16 mg/L, were prepared by adding 10 mL of HA stock solution, or stock solution diluted with 0.1M NaOH, to 90 mL of AW or PF4. Since the addition of HA, dissolved in 0.1M NaOH, to the water increased the pH, the samples were titrated with 6M HCl to reduce the pH to the initial values. Two negative controls were used, one with the addition of 0.1 NaOH instead of HA (solvent control) and another with the addition of Milli-Q water. In vitro sporulation efficiency was then determined after washing *A. astaci* and *S. parasitica* mycelia with HA-supplemented water samples using the 12-well plate protocol described above. Three biological replicates were performed for each oomycete species, water sample and HA concentration.

### 2.4. Data Analysis

To analyze the effects of different physico-chemical properties of water samples (explanatory variables or predictors, X: SAC, DOC, pH, EC, NH_4_-N, NO_3_-N, SO_4_^2−^, F^−^, Cl^−^, Mg^2+^, Ca^2+^, Br^−^, TN, TP) on *A. astaci* and *S. parasitica* sporulation efficiency (response variables, Y), Partial Least Squares Regression (PLS-R) analysis was performed using the XLSTAT version 2021.3.1.1189 software provided by Microsoft Excel by Addinsoft.

The non-parametric Mann–Whitney U-test was used to determine the significance of differences in sporulation efficiency between HA-supplemented AW and PF4 water samples. The Kruskal–Wallis test, followed by Dunn’s post hoc test, was used to estimate the significance of differences in sporulation efficiency between the different concentrations of HA and the negative controls. In all cases, the significance level was set at *p* < 0.05. The tests were performed in R v. 3.2.0 (R Core Team, 2020).

## 3. Results

**Figure 2:**
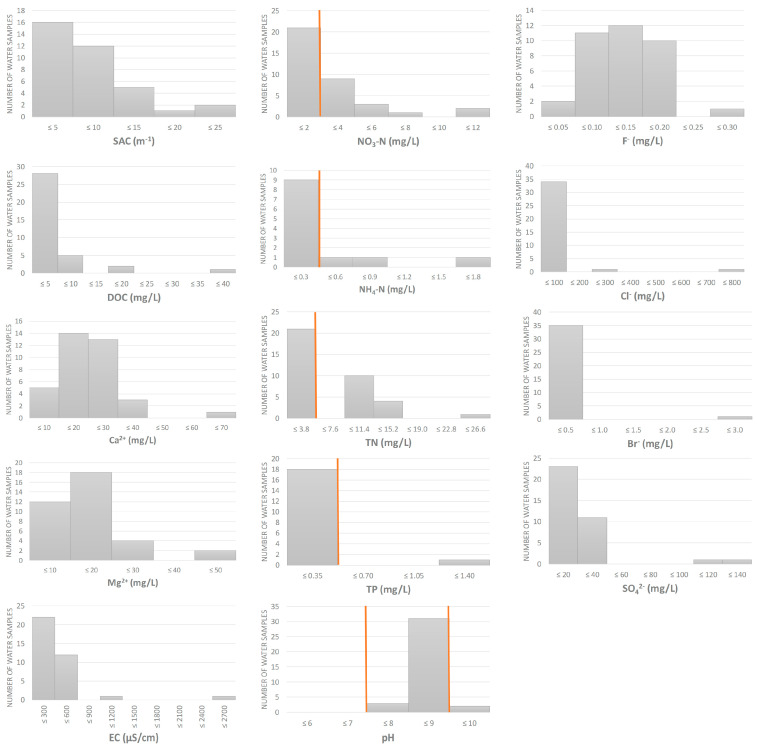
Physico-chemical properties of water samples (*n* = 36). Orange lines represent the thresholds set by national legislation, available for some of the measured parameters [45], i.e., the upper threshold for NO_3_-N, NH_4_-N, TN and TP, and lower and upper thresholds for pH. SAC = spectral absorption coefficient, DOC = dissolved organic carbon, EC = electrical conductivity, TN = total nitrogen, TP = total phosphorus.

Physico-chemical analyses showed an overall good quality of the water samples collected at 36 sites, covering different water types and biogeographical regions in Croatia (Figure 1 and Figure 2, Table S2). However, some parameters in some samples exceeded the thresholds set by national legislation [45], particularly those related to nitrogen content. Nitrate-N and total nitrogen were elevated in 15 out of 36 samples (41%), ammonium-N in 3/36 (8%), pH (>9) in 2/36 (6%) and total phosphorus in 1/36 (3%).

We used the collected water samples to wash the mycelia of oomycete pathogens *A. astaci* and *S. parasitica* and induce sporulation. The average sporulation efficiency was similar for both pathogens, i.e., 4444 zoospores/mL for *A. astaci* (min = 0, max = 42,222) and 4537 zoospores/mL for *S. parasitica* (min = 0, max = 37,778), but we obtained variable zoospore numbers upon using different water samples, as listed in Table S2.

**Figure 3:**
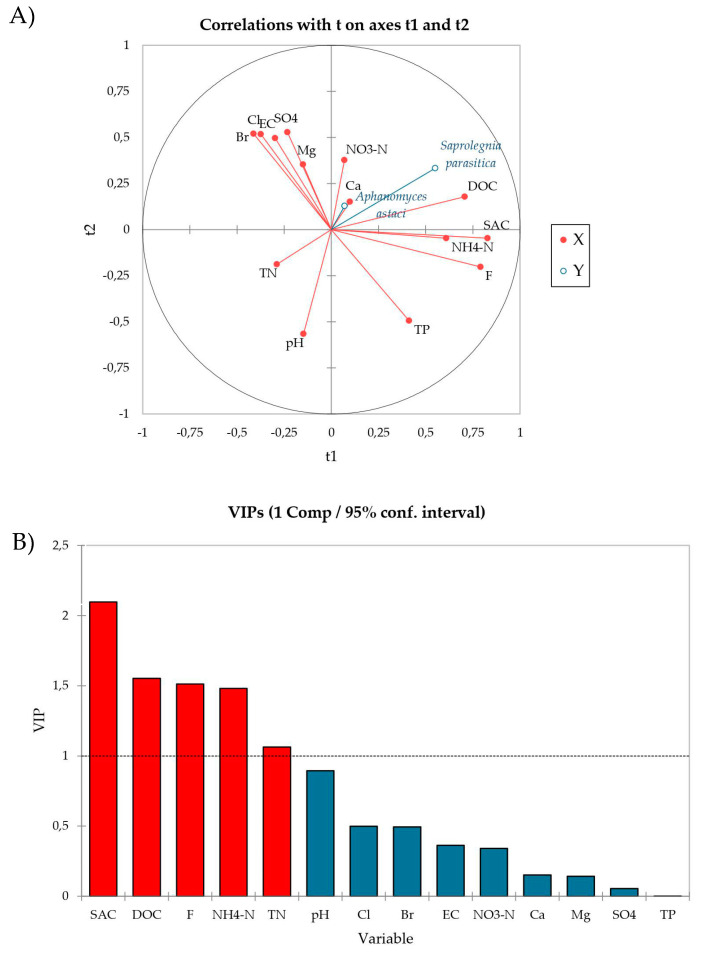
(**A**) Radar of correlation describing the relationship between the sporulation intensity of *A. astaci* and *S. parasitica* (response variable, Y, blue lines) and physico-chemical parameters of water (explanatory variables, X, red lines). The percentages of the variances in X and Y explained by each variable are indicated on the respective axes. (**B**) The Variable Importance in the Projection (VIPs) for explanatory variables of the first component (t1). VIPs > 1 indicate the explanatory variables that contribute the most to the PLS model.

Using PLS-R multivariate analysis we examined the relationship between the sporulation intensity of *A. astaci* and *S. parasitica* (response variables, Y) and the physico-chemical parameters of water (explanatory variables, X). Quality indices Q^2^(cum), R^2^Y(cum) and R^2^X(cum) of the obtained model were 0.09, 0.15 and 0.21, respectively, for the first component and −0.03, 0.22 and 0.36, respectively, for the second component. The PLS-R modelled relationship between blocks of response and explanatory variables is visually presented as a radar of correlation (Figure 3A). In the radar, positively correlated variables are presented close to each other, and those negatively correlated are located far apart, while the strength of the correlation between any two variables is predicted by their respective r values (listed in correlation matrix, Table S3). Based on this, SAC (r = 0.476), DOC (r = 0.375), NH_4_-N (r = 0.356) and F^−^ (r = 0.353) most strongly positively affected *S. parasitica* sporulation, while *A. astaci* sporulation was not significantly affected, neither positively nor negatively, by any of the analyzed water parameters (Figure 3A, Table S3). Moreover, SAC, DOC, F^−^, NH_4_-N and TN also had VIP values > 1 (Figure 3B), indicating that these parameters are relevant for explaining the sporulation efficiency and contribute significantly to the PLS model [46,47].

**Figure 4:**
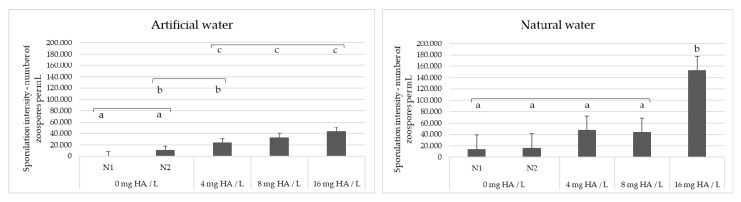
*Saprolegnia parasitica* sporulation intensity after washing the mycelium with artificial water (AW) or natural water (PF4) supplemented with increasing concentrations of humic acid (HA). In control experiments N1 and N2, water and 0.1 M NaOH were mixed with water samples instead of HA solution in 0.1 NaOH, respectively. Bars marked with the same letter (a, b or c) within each panel are not statistically different from one another.

Based on the positive effect of SAC and DOC on the sporulation intensity of *S. parasitica*, we experimentally tested whether the addition of environmentally relevant concentrations of humic acid (HA), a widespread substance that contributes to both SAC and DOC, to water can stimulate sporulation of freshwater oomycete pathogens. The water samples used, artificial water (AW) and one of the natural water samples from our dataset (PF4, Table S2), were selected on the basis that they did not induce sporulation when previously used to wash the oomycete mycelia. AW contains no organic matter (see Materials and Methods), while the SAC and DOC values of PF4 were below average: 3.39 m^−1^ and 2.73 mg/L, respectively (average SAC in our dataset was 7.09 m^−1^, while the average DOC was 5.13 mg/L). The results obtained were in agreement with the PLS model and showed that the sporulation efficiency of *S. parasitica* increased with increasing concentration of HA (4, 8 and 16 mg/L) for both AW and PF4 (Figure 4). In contrast, the addition of HA to the water had no effect on the sporulation of *A. astaci*, i.e., sporulation failed when washing the mycelia with both control samples and HA-supplemented water (data not shown). The sporulation efficiency of *S. parasitica* was significantly higher after washing the mycelium with PF4 than after washing with AW (Mann–Whitney U-test; W = 639.5; *p* = 0.004), probably due to baseline presence of organic matter in the natural water sample. In addition, the sporulation efficiency of *S. parasitica* was significantly higher in HA-supplemented water than in the negative controls, for both AW and PF4 (Kruskal–Wallis test; *p* < 0.001) (Figure 4).

## 4. Discussion

We present for the first time the differences in sporulation intensity of the freshwater oomycete pathogens *A. astaci* and *S. parasitica* in natural water samples with different physicochemical properties and show that the addition of humic acid, a widespread substance contributing to SAC and DOC levels in freshwater, can trigger sporulation of *S. parasitica*. These results are important both in terms of extending the knowledge on the ecological requirements of these pathogens and as a starting point for the optimization of laboratory protocols for the induction of sporulation.

The parameters related to organic matter in water, especially its aromatic part, were the most important factors positively affecting the sporulation intensity of *S. parasitica*. In contrast, sporulation of *A. astaci* was not significantly affected by any of the water quality parameters studied. Moreover, the sporulation intensity of *A. astaci* and *S. parasitica* was not correlated. These results are consistent with the contrasting life strategies of these two pathogens. *Aphanomyces astaci* is a specialized animal parasite with a narrow host range, whereas *S. parasitica* has a much wider host range and is usually considered an opportunistic secondary pathogen that alternates between a saprophytic and pathogenic lifestyle [4,48], although some *S. parasitica* strains have been reported to be highly virulent and cause primary infections [16,49,50,51,52]. It has already been shown that the survival of *A. astaci* depends mainly on the presence of host crayfish species [6,23], while our unpublished results on the monitoring of *S. parasitica* in freshwater systems have shown that its load is correlated with some parameters of water composition, such as electrical conductivity and calcium. We hypothesize that host-specific factors might favor sporulation of *A. astaci*, while in the case of *S. parasitica,* water composition may be more important, but further studies are needed to clarify these possible differences.

The PLS-R results showed that the sporulation intensity of *S. parasitica* was positively correlated with the SAC and DOC content of the water samples in our dataset. The DOC levels in freshwater environments vary widely from 0.1 to hundreds of mg/L, but their average values were reported to be about 5 mg/L [53,54], similar to the values measured in our dataset. Absorbance at 254 nm (SAC) usually refers to the presence of organic compounds in water, especially those containing aromatic rings or unsaturated carbon bonds (double or triple) in their molecular structure. Thus, the PLS-R results indicate that the presence of aromatic organic matter in water, such as humic acids, can promote sporulation of *S. parasitica*. This was confirmed experimentally, as washing *S. parasitica* mycelia with a series of HA-supplemented water samples stimulated sporulation compared to negative controls. In contrast, the addition of HA had no effect on the sporulation of *A. astaci*. Interestingly, humic substances were previously shown to inhibit mycelial growth of *S. parasitica* and a number of related oomycetes from the order Saprolegniales (genera *Saprolegnia*, *Achlya*, *Leptolegnia*, *Pythium*), but without concomitant inhibition of sporangia development [55,56]. This is consistent with our results and suggests that in the presence of humic substances, mycelial growth is impaired and oomycetes therefore switch to the production of sporangia and motile zoospores that can spread to new, more favorable environments.

Furthermore, the PLS-R modelling results suggest that increased fluoride and ammonium concentration could also act as a sporulation trigger, at least for *S. parasitica*. The range of fluoride concentrations in freshwater is between 0.01 and 0.3 mg/L [57], which is in accordance with the average fluoride concentration of 0.1 mg/L in our dataset. The induction of sporulation by environmentally relevant fluoride concentrations could be explained as a response to unfavorable environmental conditions, as in the case of the humic substances mentioned above. High fluoride concentrations have been shown to have negative effects on microbial physiology [58,59,60,61], but there are no data yet on the toxicity of fluoride to oomycetes. Furthermore, in some of our samples, ammonium-N concentrations were above the threshold of 0.3 mg/L set by national legislation [45]. Environmentally relevant ammonium concentrations (0.05 and 0.5 mg/L) were related to an increased susceptibility of rainbow trout (*Oncorhynchus mykiss*) to saprolegniosis [62,63]. This was explained by the host stress response and specific impairments of the defense mechanisms against saprolegniosis, but, considering our results, might also partly be due to the ammonium-induced sporulation increase and thereby the increased virulence of the pathogen. However, it was shown that in the presence of high ammonium-N concentrations (8 and 16 mg/L, not found in our dataset) the relative abundance of oomycetes in freshwater habitat can decrease [64]. Similarly, our analysis of *S. parasitica* load in water samples by droplet digital PCR (ddPCR) showed a negative correlation with ammonium and fluoride concentration (unpublished results). Additional experiments, using a series of increasing ammonium concentrations in the water, should be performed to determine the ammonium concentration range that promotes sporulation, and to compare the effects of ammonium towards mycelium and zoospores of pathogenic oomycetes.

Altogether, our results suggest that some substances might suppress the mycelial growth of the pathogen in a certain range of environmentally relevant concentrations and at the same time promote sporulation, thus facilitating the spread of the pathogens into more favorable environment. Thus, the effects of these compounds, such as ammonium and fluoride, on the sporulation intensity and virulence of freshwater oomycete pathogens should be tested, as it was tested here with HA. Furthermore, one of the limitations of this study is that we used only a single isolate from each species. This is unlikely to be representative at the species level, as significant within-species differences in mycelial growth rate, sporulation temperature, zoospore motility, and other parameters were observed for both *A. astaci* [65] and *S. parasitica* [66,67,68]. Therefore, a wider range of isolates/genotypes should be included in in vitro sporulation intensity tests in the future.

The knowledge obtained would enable, from an ecological standpoint, the prediction of the water conditions that might promote the pathogen spreading in natural environments and aquaculture facilities, and thereby aid in the development of preventive measures. On the other hand, the research community working on pathogens from the order Saprolegniales would greatly benefit if the composition of water used for sporulation would be standardized. A range of artificial water samples, with defined composition, could be designed and tested to provide optimal and reproducible oomycete sporulation in laboratory conditions.

## Data Availability

The data presented in this study are available in Tables S1 and S2.

## Supplementary Materials

The following are available at https://www.mdpi.com/article/10.3390/microorganisms10030520/s1, Table S1: Water samples collected at different locations in Croatia; Table S2: Physico-chemical parameters of the collected water samples and the average number of zoospores obtained; Table S3: Correlation matrix describing the relationship between sporulation efficiency of *A. astaci* and *S. parasitica* (response variable) and physico-chemical parameters of water (explanatory variables).
